# Effects of optogenetic stimulation of basal forebrain parvalbumin neurons on Alzheimer’s disease pathology

**DOI:** 10.1038/s41598-020-72421-9

**Published:** 2020-09-22

**Authors:** Caroline A. Wilson, Sarah Fouda, Shuzo Sakata

**Affiliations:** Strathclyde Institute of Pharmacy and Biomedical Sciences, University of Strathclyde, 161 Cathedral Street, Glasgow, G4 0RE UK

**Keywords:** Neuroscience, Alzheimer's disease

## Abstract

Neuronal activity can modify Alzheimer’s disease pathology. Overexcitation of neurons can facilitate disease progression whereas the induction of cortical gamma oscillations can reduce amyloid load and improve cognitive functions in mouse models. Although previous studies have induced cortical gamma oscillations by either optogenetic activation of cortical parvalbumin-positive (PV+) neurons or sensory stimuli, it is still unclear whether other approaches to induce gamma oscillations can also be beneficial. Here we show that optogenetic activation of PV+ neurons in the basal forebrain (BF) increases amyloid burden, rather than reducing it. We applied 40 Hz optical stimulation in the BF by expressing channelrhodopsin-2 (ChR2) in PV+ neurons of 5xFAD mice. After 1-h induction of cortical gamma oscillations over three days, we observed the increase in the concentration of amyloid-β42 in the frontal cortical region, but not amyloid-β40. Amyloid plaques were accumulated more in the medial prefrontal cortex and the septal nuclei, both of which are targets of BF PV+ neurons. These results suggest that beneficial effects of cortical gamma oscillations on Alzheimer’s disease pathology can depend on the induction mechanisms of cortical gamma oscillations.

## Introduction

Amyloid plaques are a hallmark of Alzheimer’s disease (AD) along with other pathological features^1–6^. Although a wide range of molecular targets have been identified for pharmaceutical interventions^7–9^, it is also important to develop alternative non-pharmaceutical intervention strategies given the complexity of AD pathology. One of such strategies is a neuromodulation approach^10–13^.

While abnormalities in neuronal activity have been associated with AD^14–21^, recent optogenetic or chemogenetic studies in mouse models have demonstrated that the artificial manipulation of neuronal activity can modify AD pathology^10,22,23^. For example, increased or decreased neural firing promotes or suppresses the spread of Amyloid-β (Aβ), respectively^22,23^. In particular, the artificial induction of cortical gamma oscillations is an attractive option because it can reduce amyloid load and improve cognitive functions by activating microglia^10,11,24,25^ and because optogenetic restoration of hippocampal gamma oscillations can rescue memory impairments^26^.

Although this new approach based on gamma oscillations offers an exciting opportunity, cortical gamma oscillations can be induced by multiple mechanisms^27,28^. While it is likely that different methods to induce cortical gamma oscillations (e.g., optogenetic activation or sensory stimulation) activate distinct neural ensembles, it is unclear whether any approaches to induce cortical gamma oscillations can be beneficial. Addressing this issue is important to determine the underlying molecular and cellular mechanisms of how cortical gamma oscillations can modify AD pathology.

In the present study, we focus on the basal forebrain (BF) because of the following reasons: first, the BF is one of the most affected brain regions in AD^29–31^. Second, BF neurons provide cortex-wide projections^29,32–36^. Third, the activation of BF neurons can modify cortical states^37,38^. Thus, the BF can be a unique target to modulate cortex-wide neural activity, hence AD pathology. While the BF consists of multiple nuclei and cell types^29,33,34,39^, we are particularly interested in parvalbumin-positive (PV+) neurons^35,37,40^.

BF PV+ neurons strongly innervate the pallidum, the striatum including the lateral septal complex, the anterior cingulate area, the infralimbic area, the retrosplenial area, the hippocampus, the thalamus, the lateral hypothalamus, the motor-related midbrain region and the behavioral state-related pontine region^35^. Gamma oscillations in the frontal cortex can be induced by optogenetic stimulation of BF PV+ neurons^37^. To test the hypothesis that the cortical gamma oscillations can modify AD pathology, we induced cortical gamma oscillations by optogenetically stimulating BF PV+ neurons in 5xFAD mice, the most aggressive AD mouse model^41^. Contrary to previous observations, we observed increased Aβ_1–42_ in the frontal cortical region, but not Aβ_1–40_. We also found that amyloid plaques increased in the medial prefrontal cortex and the septal nuclei. These results imply distinct molecular and cellular responses to different approaches to induce cortical gamma oscillations.

## Results

### The induction of cortical gamma oscillations by optogenetic stimulation of basal forebrain PV+ neurons

To confirm whether optogenetic stimulation of BF PV+ neurons can modulate BF and cortical population activity in vivo, we performed simultaneous recording from the BF and the neocortex (either the medial prefrontal cortex, mPFC, or the auditory cortex, AC) while optically stimulating BF PV+ neurons (Supplementary Fig. S1A). We recorded 224 BF neurons and 153 cortical neurons (113 mPFC and 40 AC neurons) from four recording sessions (2 animals). In the BF, 39.2% of recorded neurons (88/224) were significantly activated whereas 12.0% of neurons (27/224) were significantly suppressed (Supplementary Fig. S1B,C). We also found that 4.5% of cortical neurons (7/153) were significantly suppressed during optical stimulation whereas no cortical neurons were activated (Supplementary Fig. S1D,E). All suppressed cortical neurons were found in the mPFC. Out of 7 suppressed mPFC neurons, three neurons were narrow-spiking neurons, putative fast-spiking interneurons (10.3%, 3/29) whereas only 3.2% of broad-spiking neurons, putative excitatory neurons (4/124), were suppressed. Thus, optogenetic stimulation of BF PV+ neurons could modulate BF population activity and preferentially suppress cortical narrow-spiking neurons.

**Figure 1:**
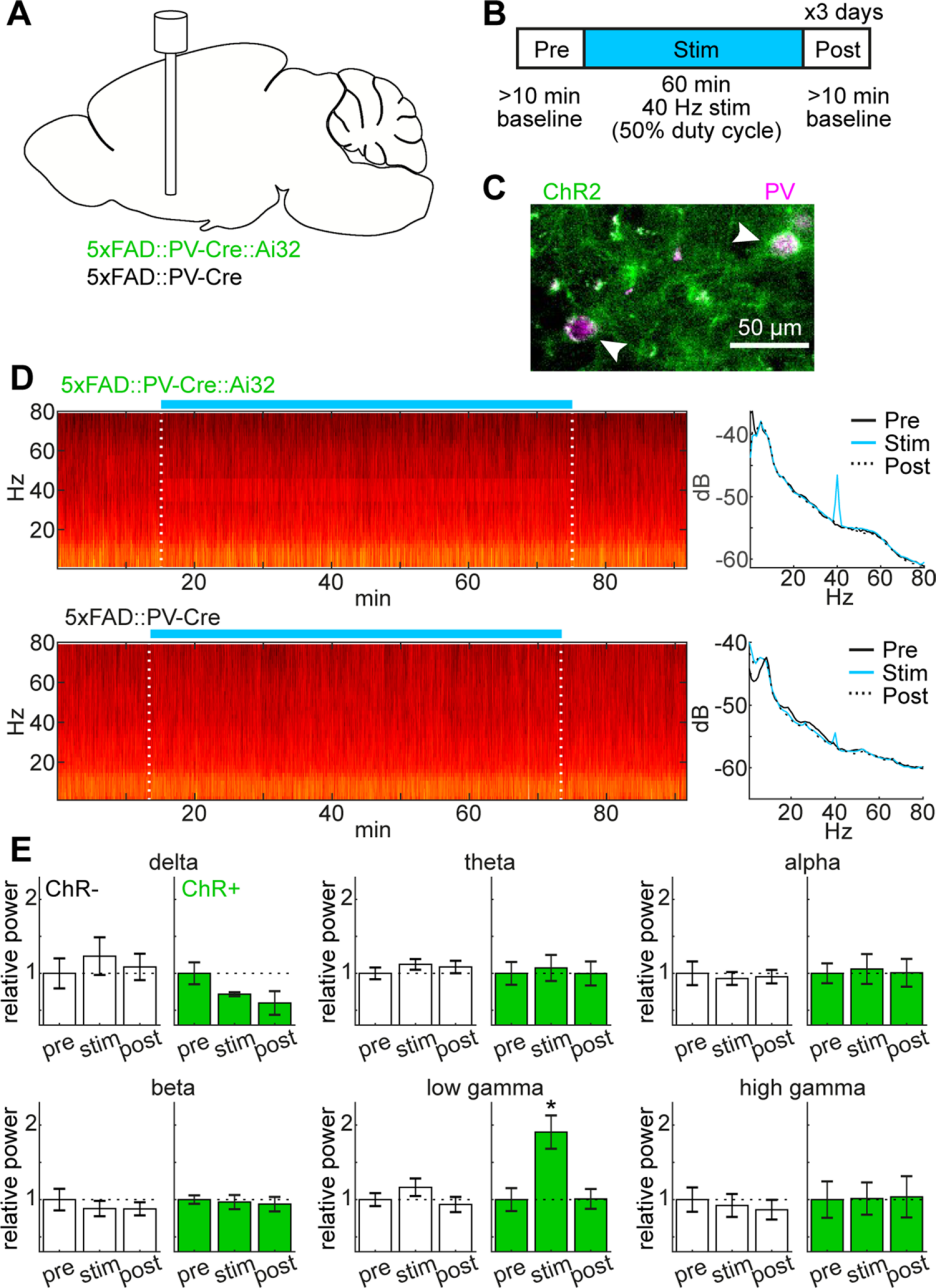
The induction of cortical gamma oscillations by optogenetic stimulation of BF PV+ neurons in 5xFAD mice. (**A**) A diagram of the experimental approach, showing the optical fiber implant in the BF and genotypes used in this study. Ai32 is a Cre-dependent ChR2 mouse. (**B**) A timeframe of the optogenetic experiment. (**C**) A photograph, showing co-expression of ChR2-EYFP (green) and PV (magenta) in the BF. ChR2 expressed in the membrane of PV+ cells. (**D**) Examples of spectrogram (*left*) and power spectral density (*right*) in ChR2+ (*top*) and ChR2− (*bottom*) animals. (**E**) Relative changes in spectral power across frequency bands in ChR2+ (*green*) and ChR2− groups (*white*). delta, 0.5–4 Hz; theta, 5–8 Hz; alpha, 8–12 Hz; beta, 15–30 Hz; low gamma, 38–43 Hz; and high gamma, 50–80 Hz. *p < 0.05 (one-way ANOVA with post-hoc HSD test).

To induce cortical gamma oscillations in an AD mouse model, we expressed ChR2 in PV+ neurons of 5xFAD mice (Fig. 1A,C) and applied 40 Hz optical stimulation for 1 h (Fig. 1B) in the BF. 5xFAD::PV-Cre mice were used as a control without expressing ChR2. This control group underwent the same surgical operations and optical stimulation. As previously reported^37^, 40 Hz optical stimulation in 5xFAD::PV-Cre::Ai32 mice elicited the strong modulation in cortical EEGs at 40 Hz (Fig. 1D). To quantify this tendency, we computed spectral power in each period (Pre, Stim, and Post periods) across the following frequency bands: delta (0.5–4 Hz), theta (5–8 Hz), alpha (8–12 Hz), beta (15–30 Hz), low gamma (38–43 Hz), and high gamma (50–80 Hz) (Fig. 1E). Although we observed a tendency of reduction in the delta power, the effect was not significant (*F*_2,6_ = 2.63, *p* = 0.15, one-way ANOVA). This tendency may be explained by the fact that activation of BF PV+ neurons leads to arousal^33,37^. We confirmed that the low gamma power increased only during optical stimulation period in ChR2 expressing animals (*F*_2,8_ = 8.91, *p* < 0.05, one-way ANOVA with post-hoc HSD test). Thus, optogenetic stimulation of BF PV+ neurons can induce cortical gamma oscillations in 5xFAD mice.

### Increased amyloid burden following optogenetic stimulation

**Figure 2:**
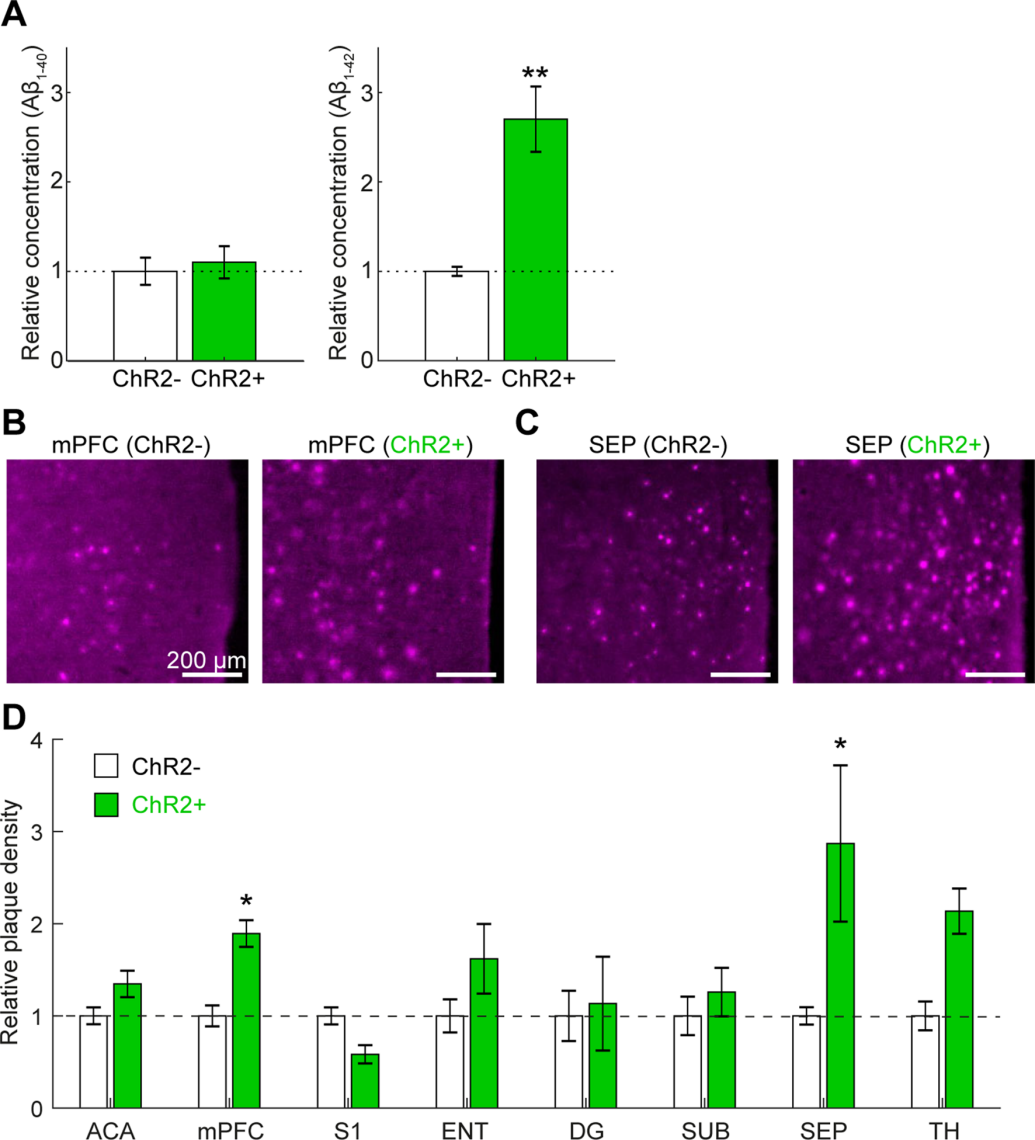
Biochemical and histological analysis of changes in amyloid load following optogenetic stimulation. (**A**) Relative concentration of Aβ_1–40_ (*left*) and Aβ_1–42_ (*right*) in the prefrontal cortical area, measured by ELISA. **p < 0.01 (*t*-test). (**B**,**C**) Photographs of thiazine-red-stained sections in the mPFC (**B**) and the SEP (**C**). (**D**) Plaque density across brain regions. The values in each brain region were normalized by the average plaque density in ChR2− group. *p* < 0.05, two-way ANOVA with post-hoc HSD test. *ACA* anterior cingulate area, *mPFC* medial prefrontal cortex, *S1* primary somatosensory cortex, *ENT* entorhinal cortex, *DG* dentate gyrus, *SUB* subiculum, *SEP* septum, *TH* ventral posteromedial nucleus of the thalamus.

To examine whether optogenetic stimulation affects AD pathology, we collected brain samples after three-day optogenetic stimulations and measured the concentration of Aβ_1-40_ and Aβ_1-42_ by performing ELISA (Fig. 2A). Because BF PV+ neurons project primarily to the prefrontal cortical regions^35^, we analyzed amyloid load in the prefrontal cortical area and the BF separately. Compared to the control (ChR2−) group, we found significant increase in the concentration of Aβ_1–42_ in the prefrontal cortical area (*p* < 0.01, *t*-test) and the BF (1.00 ± 0.12 in ChR2−, 2.4 ± 0.48 in ChR2+ , *p* < 0.05, *t*-test) whereas we did not find any significant changes in prefrontal Aβ_1–40_ (*p* = 0.69, *t*-test) and BF Aβ_1–40_ (1.00 ± 0.02 in ChR2−, 1.02. ± 0.06 in ChR2+ , *p* = 0.74, *t*-test).

To verify these observations, we also performed histological analysis across multiple brain regions (Fig. 2B–D, Supplementary Figs. S2, S3). We analyzed the anterior cingulate area (ACA), the medial prefrontal cortex (mPFC) including the prelimbic and infralimbic areas, the primary somatosensory cortex (S1), the entorhinal cortex (ENT), the dentate gyrus (DG), the subiculum (SUB), the septum (SEP), and the ventral posteromedial nucleus of the thalamus (TH). In addition, we also examined the CA1 and CA3 in the hippocampus (Supplementary Fig. S3). We found that the mPFC (Fig. 2B) and the SEP (Fig. 2C) exhibit significant increase in the number of amyloid plaques in ChR2+ groups (*F*_7,123_ = 2.60, *p* < 0.05, two-way ANOVA with post-hoc HSD test) (Fig. 2D). These results indicate that the induction of cortical gamma oscillations by optogenetically stimulating BF PV+ neurons increases amyloid load in several brain regions.

## Discussion

The induction of cortical gamma oscillations can modify AD pathology. Because cortical gamma oscillations typically arise locally^28^, other means to induce *cortex-wide* gamma oscillations would be ideal for a future treatment. In addition, because gamma oscillations can be induced by several distinct mechanisms^27,28^, it is important to evaluate whether different approaches to induce cortical gamma oscillations can also be beneficial. We thought that the BF PV+ neurons can be an ideal target to address these issues. Here we found that optogenetic activation of BF PV+ neurons in 5xFAD mice increases amyloid load in several brain regions, including the mPFC and the septal nuclei. Our results suggest that effects of cortical gamma oscillations on AD pathology can depend on their induction mechanisms.

Previous studies showed that optogenetic activation of BF PV+ neurons can induce cortical gamma oscillations and arousal^33,37^. We confirmed the optogenetic induction of gamma oscillations in 5xFAD mice (Fig. 1). Although we were initially concerned that the sharp increase in low gamma power might have been due to an optical artifact, we did not observe a similar modulation in the control group, indicating that the increase in low gamma power can be explained by the expression of ChR2. We also observed a tendency of the reduction in delta power. This observation is also consistent with the notion that the activation of BF PV+ neurons induce arousal^33^.

While BF PV+ neurons project to a wide range of brain regions, the mPFC and the septum are also target regions^35^. Our results are consistent with this anatomical observation in the sense that optogenetic activation of BF PV+ neurons can modify their downstream neurons. Because we took a transgenic approach where a Cre-dependent optogenetic mouse line (i.e., Ai32) was crossed with PV-IRES-Cre mice, most of PV+ neurons express ChR2. The advantage of this transgenic approach is the consistency of ChR2 expression across animals. On the other hand, a viral approach can target a specific brain region while this approach may introduce experimental variability with respect to injection sites and expression level. Because the previous study^10^ optogenetically targeted the hippocampus, but not neocortical areas, it would be important to confirm that optogenetic activation of neocortical PV+ neurons can also reduce amyloid load. Another limitation of the present study is the moderate statistical power (0.67) in the ELISA results (Fig. 2A). In a future large study, it would also be important to investigate sex differences because gender plays a role in AD pathology in both humans and animal models^42–45^.

We initially expected the decrease in amyloid load after optogenetic stimulation of BF PV+ cells because previous studies show that the induction of cortical gamma oscillations can reduce amyloid burden and improve cognitive functions^10,24,25^. What can explain this discrepancy? One difference from the original study^10^ is animal age. In the present study, we used 4–6 months old mice whereas the original study used 3 months old mice^10^. However, because the subsequent study^24^ demonstrated beneficial effects in 6 months old mice, the results from our study are likely to be explained by other factors, rather than age. Another difference is the method to induce cortical gamma oscillations. Whereas previous studies induced gamma oscillations by either optogenetic stimulation of hippocampal PV+ neurons or 40 Hz sensory stimulus^10,24^, we induced cortical gamma oscillations by activating BF PV+ neurons. Because BF GABAergic neurons preferentially target cortical interneurons^46^, our approach could suppress cortical PV+ neurons, rather than activating them. Indeed, our results (Supplementary Fig. S1) support this notion. Because cortical gamma oscillations can be induced by multiple mechanisms^27,28^, it is possible that these methodological differences may induce distinct molecular and cellular responses, resulting in opposing effects on AD pathology. In addition, because microglia plays a key role in AD pathogenesis^3,5,6^ and microglia exhibits highly dynamic responses depending on global brain states^47,48^, it is also conceivable that the optogenetic activation of BF PV+ neurons induces not just cortical gamma oscillations, but also changes in neuromodulatory tones, leading to microglial responses in the cortex different from other approaches.

Although our finding regarding the modification of Aβ_1–42_ (Fig. 2A) is consistent with another optogenetic study^22^, it remains to be determined how Aβ_1–42_ was selectively increased. While both forms of Aβs differ in their metabolism and aggregation mechanisms^49^ and the cerebrospinal fluid Aβ_1–42_/Aβ_1–40_ ratio has been considered as a diagnostic marker of AD^50^, further investigation on activity-dependent regulation of Aβs will provide insight into the pathogenesis of AD.

It would be crucial in the future to determine whether optogenetic stimulation of BF PV+ neurons could also modify cognitive functions. A recent study showed that the acute optogenetic induction of low gamma oscillations (but not high gamma) in the hippocampus can rescue memory impairments^26^. Although we did not observe any significant changes in plaque density in the hippocampus, hippocampus-dependent functions may be modified through the modulation of septal activity. Thus, our current results do not rule out the possibility that our optogenetic approach can still be beneficial for a certain cognitive function.

In summary, 40 Hz optogenetic activation of BF PV+ neurons in 5xFAD mice increases amyloid load. Although the induction of cortical gamma oscillations is still a promising approach to modify AD pathology, the outcomes can vary depending on how to induce gamma oscillations. A better understanding of underlying circuit mechanisms of gamma oscillations as well as molecular and cellular responses to distinct gamma oscillations is crucial to fully implement this novel approach for treatment and prevention of AD and other neurodegenerative diseases.

## Methods

### Animals

All animal experiments were performed in accordance with the United Kingdom Animals (Scientific Procedures) Act of 1986 Home Office regulations and approved by the University of Strathclyde Animal Welfare and Ethical Review Body and the Home Office (PPL70/8883). 5xFAD mice (JAX006554)^41^ were crossed with PV-IRES-Cre mice (JAX008069), followed by crossing with Ai32 mice (JAX012569). All genotyping was performed by Transnetyx using real-time PCR. Six 4–6 month old animals in both sexes were used in this study: three 5xFAD::PV-Cre::Ai32 mice (1 male, 2 females) and three 5xFAD::PV-Cre (2 males and 1 female). Two PV-Cre::Ai32 mice (female, 4 months old) were used for simultaneous recording of the BF and cortical population activity while optogeneticially stimulating BF PV+ neurons (Supplementary Fig. S1). All animals were housed individually in high-roofed cages with a 12 h:12 h light/dark cycle (light on hours: 7:00–19:00). They had ad libitum access to food and water. All experiments were performed during the light period.

### Surgery

The surgical procedures and in vivo electrophysiological recording procedures were similar to previous studies^51–53^. Mice were anesthetized with isoflurane (5% for induction, 1–2% for maintenance) and placed in a stereotaxic apparatus (SR-5M-HT, Narishige). Body temperature was maintained at 37 °C with a feedback temperature controller (50-7221-F, Harvard bioscience). Lidocaine (2%, 0.1 mL) was administered subcutaneously at the site of incision. Carprofen (Rimadyl, 5 mg/kg) was also administered subcutaneously at the back. After incision, the skull was exposed and cleaned. Four bone screws were implanted on the skull for monitoring cortical electroencephalograms (EEGs) (coordinate for frontal EEGs: AP, 1.5 mm; ML, ± 1 mm) (coordinate for parietal EEGs: AP, − 2 mm; ML, ± 2 mm). An additional bone screw was implanted over the cerebellum as a ground/reference. These screws were connected to a two-by-three connector (SLD-112-T-12, Samtec). An optical fiber canula (CFM14L05, Thorlabs) was implanted to target the basal forebrain (AP, 0 mm; ML, 1.6 mm; DV, 5 mm from the cortical surface) and fixed with dental cement. Because the implanted hemisphere was used for ELISA (see below), the exact position of the optical fiber canula could not be determined histologically. After a recovery period (at least 5 days), mice were habituated to an open field (21.5 cm × 47 cm × 20 cm) by connecting a 16-channel amplifier board (RHD2132, Intan Technologies) and an interface cable as well as a patch cable (OPT/PC-FC-LCF-200/230-1.0L, Plexon).

### Electrophysiological recording and optogenetic stimulation

Electrophysiological signals were amplified relative to a cerebellar bone screw and were digitized at 1 kHz (RHD2132 and RHD2000, Intan Technologies). These signals were fed into a data acquisition device (NI-USB-6002, National Instruments), which was controlled by custom-made LabVIEW code running on a PC. The recording was begun by > 10 min baseline recording (Pre), followed by 1-h optical stimulation (Stim). Pulses of blue light (470 nm, PlexBright, Plexon) were delivered at 40 Hz (50% duty cycles) for one hour through the patch cable attached to the implanted optical fiber. The light output at tip of the fiber was 114–178 mW/mm^2^. The recording was finished by another > 10 min baseline recording (Post). The same procedure was repeated over three consecutive days.

### Tissue collection

After the final recording session, mice were deeply anesthetized with mixture of pentobarbital and lidocaine and perfused transcardially with saline. The brains were removed immediately and trimmed into two hemispheres. One of which was used for enzyme-linked immunosorbent assays (ELISA) whereas the remaining hemisphere was immersed overnight in 4% paraformaldehyde/0.1 M phosphate buffer, pH 7.4, at 4 °C for histological analysis. For ELISA, the prefrontal cortex and basal forebrain were isolated, immediately frozen and stored at − 80 °C until use.

### ELISA

Frozen tissue samples were homogenized in phosphate buffer saline (PBS) containing protease and phosphatase inhibitor cocktails (78440, ThermoFisher) and centrifuged at 2000 rpm for 5 min. The supernatants were then transferred to new tubes and centrifuged at 13,000 rpm for 15 min. The supernatants were collected and subjected to Aβ measurement with the use of mouse Aβ_1–40_ (KMB3481, ThermoFisher) or Aβ_1–40_ ELISA kit (KMB3441, ThermoFisher).

### Histology

The fixed brain tissue was immersed in a 30% sucrose in PBS at 4 °C. The brains were frozen and were cut into coronal sections with a sliding microtome (SM2010R, Leica) with a thickness of 50 µm. Sections were stained for either PV or plaques. For PV staining, sections were washed with PBS for 5 min, three times at room temperature (RT) and then incubated in a blocking solution (10% normal goat serum, NGS, in 0.3% Triton-X in PBS, PBST) for 1 h at RT followed by incubating with primary antibodies (anti-PV, 1:1,000–2,000, P3088, Sigma-Aldrich) in 3% NGS in PBST at 4 °C overnight. After washing, sections were incubated with secondary antibodies (goat anti-mouse Alexa Fluor 594, 1:1,000, A-11005, ThermoFisher) for 2 h at RT. For plaques staining, sections were washed with PBS for 5 min, three times at RT and then incubated with Thiazine Red (0.05%, cat#27419.123, VWR) for 20 min. After washing, sections were counter-stained with DAPI (1:1,000), mounted on gelatin-coated slides and cover-slipped. Epifluorescence images were captured at × 4 magnification with a digital CMOS camera (C11440-36U, Hamamatsu) using freely available software (WinFluor). Captured brain regions were the anterior cingulate area (ACA), the medial prefrontal cortex (mPFC) including the prelimbic and infralimbic areas, the primary somatosensory cortex (S1), the entorhinal cortex (ENT), the CA1, the CA3, the dentate gyrus (DG), the subiculum (SUB), the septal nuclei (SEP) including both the lateral and medial septum, and the ventral posteromedial nucleus of the thalamus (TH). To verify whether Thiazine Red can stain plaques, some sections were used for additional experiments (Supplementary Fig. S2): for co-staining with Thiazine Red and Thioflavin S, sections were incubated with a mixture of Thiazine Red (0.05%) and Thioflavin S (0.01%, T1892, Sigma) for 20 min. For co-staining with Thiazine Red and anti-Iba1, the procedures were the same as PV staining except using primary antibodies against Iba1 (1:2000, ab54210, Abcam).

### Optogenetic and electrophysiological experiments with silicon probes

The procedures for in vivo optogenetic and electrophysiological experiments with silicon probes (Supplementary Fig. S1) were the same as previously reported^54,55^. Briefly, animals were anesthetized with isoflurane (5% induction, 1–1.5% maintenance). Lidocaine (2%, 0.1–0.3 mg) was administered subcutaneously at the site of incision. Two frontal bone screws (AP + 3 mm, ML 2 mm from bregma) were implanted, one of which was used for EEG recording. Another two screws were implanted on the cerebellum, one of which was used for a ground and a reference. A pair of nuts was attached on the skull with dental cement as a head-post. After the surgery, analgesia, Carprofen (5 mg/kg), was administered intraperitoneally. After at least 5 day recovery period, the animals were placed in a head-fixed apparatus (SR-8N-S, Narishige), with holding the head-post securely and placing the animal into an acrylic tube. This habituation procedure was continued for at least 5 days, during which the duration of the head-fixed condition was gradually extended from 15 to 60 min. A day after this habituation period, the animals were anesthetized with isoflurane (5% induction, 1–1.5% maintenance) and a craniotomy to access the BF (AP − 0.5 to − 1 mm, ML + 1.5 to + 2 mm from bregma) and the cortex (either the mPFC or auditory cortex) (for the mPFC, AP + 2 mm, ML 0 mm from bregma; for the AC, AP − 2 to − 4 mm, ML 4 to 4.5 mm from bregma) was performed. In the following day, the animals were placed in the head-fixed condition to carry out the optogenetic and electrophysiological experiments.

All experiments were performed in a single-walled acoustic chamber (MAC-3, IAC Acoustics) with the interior covered by 3 inches of acoustic absorption foam. After the surgical procedures described above, a 32 channel silicon-based optrode (A1 × 32-Poly2-10 mm-50s-177-A32OA, NeuroNexus Technologies) was inserted slowly (2–5 µm/s) using a motorized manipulator (DMA-1511, Narishige) into the BF (4.0–5.0 mm from the cortical surface). Another 32 channel silicon probe (A1×32-10mm-50-177-A32) was inserted using a manual micromanipulator (SM-25A, Narishige) for cortical recording. Broadband neural signals were amplified (RHD2132, Intan Technologies) and digitized at 20 kHz (RHD2132 and RHD2000, Intan Technologies). Optical stimulation was applied during the probe penetration to identify PV+ neurons. The recording session was initiated > 30 min after signal stabilization. For optogenetic stimulation, blue light pulses (50 ms on – 450 ms off, 470 nm, ~ 59.8 mW/mm^2^) (PlexBright, Plexon) were delivered through an optical fiber on the silicon probe. After experiments, animals were perfused transcardinally with physiological saline followed by 4% paraformaldehyde/0.1 M PBS, pH 7.4.

### Data analysis

For spectral analysis of EEGs, MATLAB (Mathworks) was used with Signal Processing Toolbox and Chronux Toolbox (https://chronux.org/). To assess the changes in spectral power at a certain frequency band, power spectral density (PSD) for each period (Pre, Stim, or Post period) was estimated with Welch’s method and the sum of PSD at the frequency band was computed. The summed PSD was further normalized by the summed PSD in Pre period for comparisons.

For spike train analysis (Supplementary Fig. S1), spike sorting was performed with freely available software (Klusta, KlustaViewa, KiloSort2, phy2). Single units with ≥ 20 isolation distance were further analyzed. To classify cortical cells, the trough-to-peak duration of average spike waveforms was measured and 3.5 ms was set as a threshold to distinguish between narrow-spiking and broad-spiking cells. To assess firing rate modulation during optical stimulation, spike counts during stimulation periods were compared with those in a pre-stimulus time window (50 ms) by using rank sum test. A *p*-value of less than 0.005 was recognized as being statistically significant.

For image analysis, a region of interest for each brain region (see above) was manually determined with Fiji and processed with a custom-written MATLAB code. The number of plaques was automatically quantified as follows: first, each image was binarized by computing a threshold, which was defined as *M* + 2.8 × *D*, where *M* is the median of pixel intensities and *D* is the average absolute deviation of pixel intensities. After applying a median filter, detected particles with a pre-determined range of sizes were recognized as plaques. Finally, the density of plaques was computed.

### Statistics

Data was presented as mean ± SEM. Statistical analyses were performed with MATLAB. Student’s *t*-test was performed in Fig. 2A. One-way ANOVA and two-way ANOVA with post-hoc Tukey’s Honest Significant Difference (HSD) test were performed in Figs. 1E, 2D, respectively.

## Supplementary information

Supplementary information.

## Data Availability

The data and codes for this article can be found at https://doi.org/10.15129/b13d7b6d-a7a1-407f-a29f-f40fdf29a6e8 and https://github.com/Sakata-Lab/PlaqueDetect.
