# Supplementary material for: Effects of optogenetic stimulation of basal forebrain parvalbumin neurons on Alzheimer’s disease pathology
Source: Sci Rep. 2020 Sep 22;10:15456. doi: 10.1038/s41598-020-72421-9 (PMC7508947; doi:10.1038/s41598-020-72421-9)
Supplement: Supplementary file 1 — Supplementary information. [file 41598_2020_72421_MOESM1_ESM.pdf]

Supplementary Information

Effects of optogenetic stimulation of basal forebrain parvalbumin neurons on Alzheimer's disease pathology

Caroline A Wilson, Sarah Fouda, Shuzo Sakata

Strathclyde Institute of Pharmacy and Biomedical Sciences, University of Strathclyde

161 Cathedral Street, Glasgow G4 0RE, UK

correspondence: shuzo.sakata@strath.ac.uk

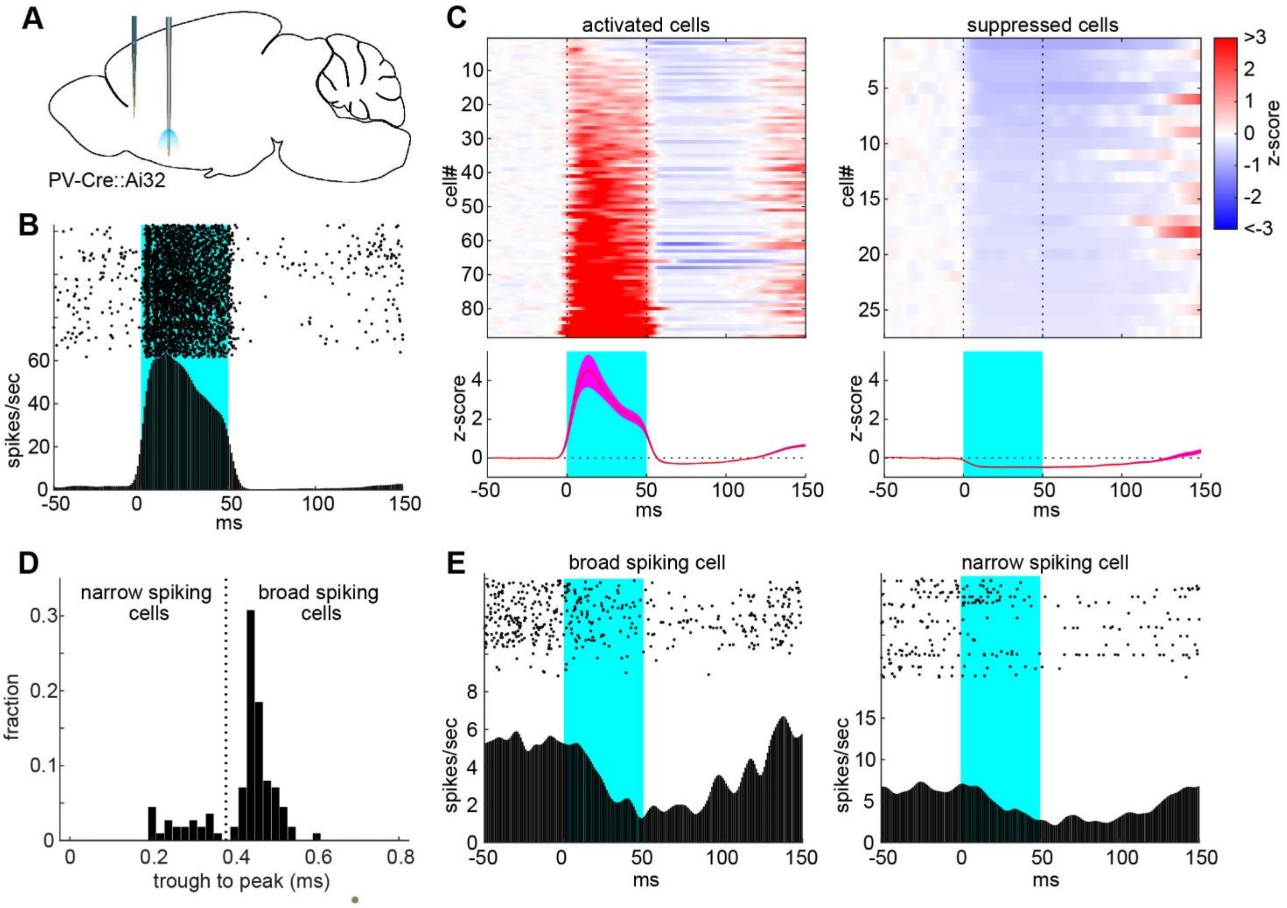

Supplementary Figure 1. Effects of optogenetic activation of basal forebrain (BF) PV+ neurons on neural responses in the BF and the cortex.

(**A**) A schematic drawing of the experimental approach. PV-Cre::Ai32 mice were used. An optrode (a 32 channel silicon probe with an optical fiber) and a 32 channel silicon probe were inserted into the BF and either the medial prefrontal cortex or the auditory cortex, respectively. 50-ms blue light pulses were applied in a head-fixed awake condition.

(**B**) An example of optogenetically activated BF cells.

(**C**) Neurons significantly activated (*left*) and suppressed (*right*) during optical stimulation. The mean response profile of each cell was z-scored based on its baseline activity. Bottom panels show average response profiles of the z-scored responses. Errors indicate SEM.

(**D**) The distribution of trough to peak duration of all cortical cells (n = 153). Broad- and narrow-spiking cells were presumed as excitatory and fast-spiking interneurons, respectively.

(**E**) Examples of cortical neurons modulated by optogenetic stimulation.

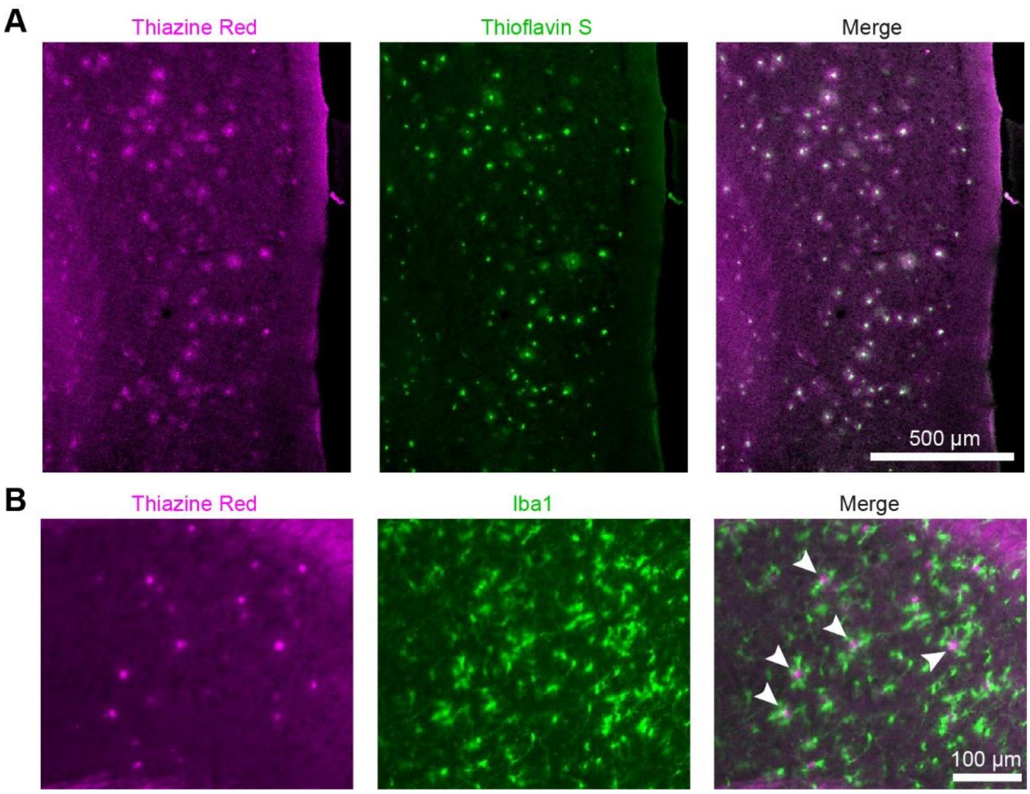

Supplementary Figure 2. Verification of Thiazine Red staining to assess Alzheimer's disease pathology.

(**A**) Co-staining with Thiazine Red (*left*) and Thioflavin S (*middle*).

(**B**) Co-staining with Thiazine Red (*left*) and anti-Iba1 (*middle*). Iba1+ cells (microglia) are accumulated around Thiazine Red positive plaques (*arrow heads*).

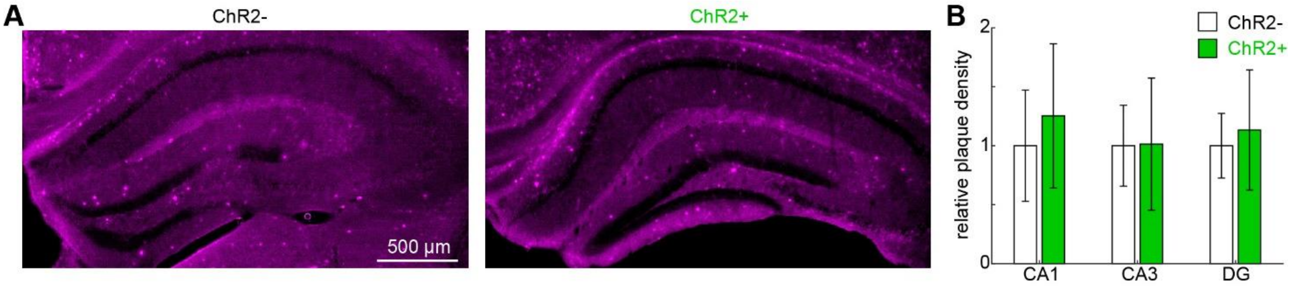

Supplementary Figure 3. Histological analysis in the hippocampus.

(**A**) Hippocampal sections stained with Thiazine Red.

(**B**) Relative plaque density across hippocampal subregions. Plaque density was normalized by the average density in the control (ChR2-) group. No statistically significant difference was detected ($F_{2,35}$ = 0.023, p = 0.97, two-way ANOVA).
